# Reduced Anxiety and Depression and Improved Mood in Older Adults Living in Care Homes After Participating in Chair Yoga

**DOI:** 10.1177/07334648241241298

**Published:** 2024-03-24

**Authors:** Keeley Frampton, Liz Oppedijk, Rebecca Hadley, Lucy E. Annett

**Affiliations:** 1 Department of Psychology, Sport and Geography, 3769 University of Hertfordshire, Hatfield, UK; 2 Accessible Chair Yoga, St Albans, UK

**Keywords:** yoga, care home, older adults, anxiety, depression, dementia

## Abstract

Providing opportunities for older adults to engage in physical and mental activity is important to support healthy aging. The present preliminary study investigated the feasibility of accessible chair yoga for older adults in care homes. Chair yoga participants (*n* = 17) were assessed before and after attending twice weekly chair yoga sessions for 8 weeks, while control participants (*n* = 16) underwent the assessments only. Participant ages ranged from 80 to 101 years and included those living with mild to severe dementia. Anxiety and depression measured by the Hospital Anxiety and Depression Scale (HADS), and negative affect measured by the Positive and Negative Affect Schedule (PANAS), improved in the chair yoga but not the control group. Balance confidence (Modified Falls Efficacy Scale) and Health-related Quality of Life (EQ-5D-3L) were unchanged. Chair yoga is a feasible activity for older care home residents, including those living with dementia, with the potential to improve mental well-being.

**What this paper adds**

• Taking part in accessible chair yoga delivered in care homes is feasible for older people resident in those care homes.
• Participation in accessible chair yoga sessions may benefit the mental health of older people resident in care homes.
• Care home residents who have dementia can engage with and benefit from accessible chair yoga sessions.

**Applications of study findings**

• The study findings can be applied to the real world setting of residential care homes, demonstrating to managers of activity programs that older residents in their care can engage with and benefit from accessible chair yoga.
• The findings will inform the design of future research studies that will be required to determine the mechanisms and longevity of the present study findings.

As the world population ages, strategies to support healthy aging are increasingly important. Engaging in physical activity is associated with lower mortality (LaMonte et al., 2018) and has been shown to contribute to successful healthy aging (Jadczak et al., 2018; Tam et al., 2022). Moreover, even light-intensity physical activity can have beneficial effects and may be useful in older individuals not able to undertake intense activity (Fuzeki et al., 2017). Inactivity is a major issue in care homes where barriers to undertaking physical exercise can include the health of the individual, lack of space, staffing, and funding constraints (Benjamin et al., 2014). Older adults classed as frail are at risk of adverse outcomes including falls, depression, cognitive decline, and hospitalization, contributing to increased health care costs (Hoogendijk et al., 2019). Mental wellbeing is also a major issue, with 60% of care home residents estimated to have mental health problems, including over 40% experiencing depression (Royal College of Psychiatrists, 2018). Participating in physical activity can improve outcomes for frail people (Clegg et al., 2012; Jadczak et al., 2018; Peyrusqué et al., 2023). A challenge for care homes is how best to provide opportunities for activities that older people, including those living with dementia, will engage with to support their physical and mental health.

Yoga is part of an ancient meditative practice originating in India. It traditionally incorporates breath work, physical postures, relaxation and meditation. Benefits of yoga for healthy older people have been reported for a range of functions, including cardiovascular health (Barrows et al., 2016), balance and mobility (Sivaramakrishnan et al., 2019), cognition (Hariprasad et al., 2013), depression (Cramer et al., 2013), and quality of life (Oken et al., 2006; Tulloch et al., 2018). Recent reviews and meta-analyses concluded that yoga and related mind-body therapies can have positive effects on cognition and depression in people with mild cognitive impairment and dementia (Bhattacharyya et al., 2021; Brenes et al., 2019; Karmacoska et al., 2023; Wang et al., 2022). However, limitations of the research were acknowledged, including heterogeneous populations, interventions, and measures (Bouega, 2020).

While the evidence that yoga can benefit older people is promising, most research has involved participants living in the community, not in care homes. Conducting research in care homes has various challenges and barriers (Lam et al., 2018). Moreover, the image that yoga is for young able-bodied people may be why care homes have not widely adopted yoga as an activity. However, yoga can be made accessible by adapting it to be practiced while seated in a chair (Playhay, 2018). Improvements in balance, mobility, activities of daily living, and reduced fear of falling, have been reported in studies employing chair yoga for participants living in the community or assisted-living homes (Furtado et al., 2016; Galantino et al., 2012; Litchke et al., 2012; McCaffrey et al., 2014). Relatively few studies have employed yoga interventions delivered in the care homes where the participants are resident (Fan & Chen, 2011; Hariprasad et al., 2013; Saravanakumar et al., 2014).

In summary, while there is evidence that yoga is a useful activity to support the physical and mental health of older people, evidence is limited regarding yoga delivered for residents in care homes. The purpose of the present study was to explore the feasibility and gather preliminary evidence regarding potential effects of delivering accessible chair yoga in care homes for residents, including those living with dementia. Twice weekly group chair yoga sessions were delivered over 8 weeks in 2 care homes, with measures taken before and after the 8 weeks. Control data was collected from residents in 2 further care homes where the chair yoga was not offered. Improvements were predicted on measures of depression, anxiety, mood, fear of falling, and health-related quality of life (HRQoL) in the chair yoga compared with the control participants.

## Methods

### Participants

Participants were recruited from 4 residential care homes in the county of Hertfordshire, UK that provided personal care for older adults who needed help with daily tasks such as washing, dressing, or eating, but not nursing care. The term “care home” in the UK refers to establishments either with or without onsite nursing provision.

Participants in 2 of the care homes took part in the chair yoga classes and assessments, while participants in the other 2 care homes underwent the assessments only and acted as a control group. This cluster design was used to avoid the risk of disaffection if control participants realized chair yoga classes were taking place in their care home to which they were not invited. Approval for the study was granted by the Health, Science, Engineering, and Technology Ethics Committee with Delegated Authority at the University of Hertfordshire. Residents were included if they lived permanently in the care home. Residents were excluded if they were bed bound, incapacitated due to illness, or did not have mental capacity and no personal consultee available. Care home staff helped identify eligible residents. Participants with mental capacity provided written informed consent. Recruitment of residents who lacked mental capacity involved personal consultees (20 of 48 participants). Personal consultee was defined as the Lasting Power of Attorney for Health and Welfare who could advise and provide written consent regarding the resident’s wishes to participate in the study.

### Measures

Demographic information collected included age, gender, time spent active in a typical week, and previous experience of yoga. The Addenbrooke’s Cognitive Evaluation (ACE III, version A) was used to assess cognitive ability (Mathuranath et al., 2000). The purpose was to characterize the cognitive status of participants, not as an outcome measure.

The Hospital Anxiety and Depression Scale (HADS) was used to assess symptoms of anxiety and depression (Zigmond & Snaith, 1994). The HADS consists of 14 questions, 7 related to anxiety and 7 to depression, with participants indicating how they felt over the preceding week on a Likert scale from 0–4, higher scores indicating more anxiety/depression.

The Positive and Negative Affect Scale (PANAS) was used to assess the participants’ general affect (Watson et al., 1988). The PANAS measures both positive and negative affect, consisting of 10 positive and 10 negative items, the participant indicating on a 1–5 Likert scale how much they identify with those states in the present moment. The higher the negative affect score, the more negative the mood; the higher the positive affect score, the more positive the mood.

Balance confidence was measured using the Modified Falls Efficacy Scale (MFS) (Hill et al., 1996). Participants reported how confident they would be carrying out 14 activities of daily living on a scale of 1–10 (with 10 being the most confident) without losing their balance. The total score was converted to an average to give an overall score.

HRQoL was measured using the EQ-5D-3L (EuroQol, 2022). This widely used instrument consists of 5 questions (on mobility, self-care, usual activities, pain/discomfort, anxiety/depression) to which participants indicate their health status on a scale from 1 to 3 (no problem, some problems, extreme problems). A summary index score was calculated applying the formula described by Dolan (1997). Participants also rated their perceived health on that day from 0–100 on a visual analog scale (EQ-5D-3L VAS).

### Chair Yoga Class Content

Chair yoga participants attended 30–45 minutes chair yoga sessions twice weekly for 8 weeks. Lakshmi Voelker Chair Yoga™ (LVCY) was used, which is particularly suited to care home residents as it uses the chair instead of the traditional mat. Poses are performed seated on the chair, rather than using the chair as a prop, with several options for each yoga pose practiced, participants choosing the level of pose that suits them. The movements are thus accessible to everyone, regardless of age or ability. Participants were encouraged to do what they could but not to over-exert themselves to ensure safety and inclusion. Props, such as cushions, yoga blocks, and footrests, were used where necessary as support. Qualified yoga teachers trained in the specialist LVCY method delivered the sessions. The same chair yoga sequence was used across the 8 weeks (Table 1).

**Table 1.** Chair Yoga Sequence of Activities During Each Session.

Sequence
1. Introduction
2. Sitting mountain: Foundational pose, encourages postural alignment
3. Deep abdominal breathing: Technique training
4. Acupressure hand movements: Warm up sequence
5. Five chair yoga poses: Varying levels of flexibility
6. Kirtan Kriya: Singing exercise based on a Kundalini yoga technique
7. Acupressure knee strengthening movements
8. Dynamic movements of the feet, hands, shoulders, elbows, wrists, and fingers
9. A chair yoga dance for dynamic movement of the hips and cross body movements
10. Return to conscious abdominal breathing as a cool down
11. Guided meditation
12. Closing sequence: Sitting mountain pose and an affirmation

**Figure 1.**
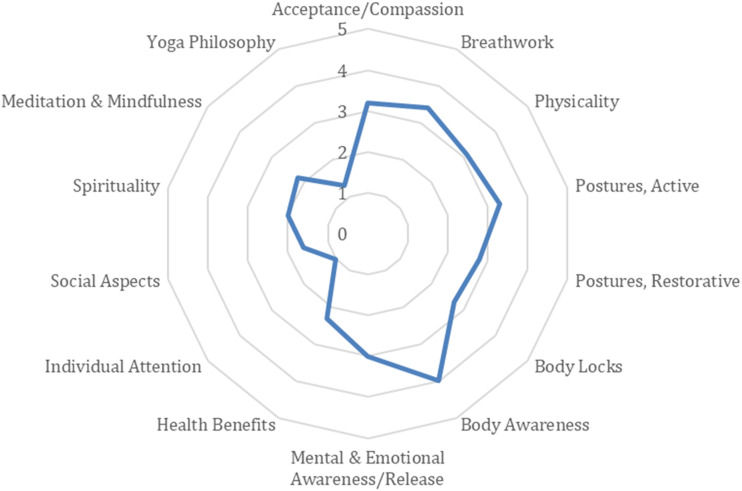
Essential properties of the chair yoga. Essential Properties of Yoga Questionnaire (EPYQ) analysis of the Lakshmi Voelker Chair Yoga™ (LVCY) series used in the study.

To facilitate comparison with other studies, the Essential Properties of Yoga Questionnaire (EPYQ, Park et al., 2018) was used by a yoga therapist independent of the study to classify the chair yoga intervention from a recording. The classification showed a larger focus on the physical aspects of yoga such as Active Postures, Breathwork, Physicality, Body Awareness, Mental and Emotional Awareness/Release, and less focus on the Spiritual Side, Meditation and Mindfulness, and Social Aspects (Figure 1).

### Procedure

Data collection was undertaken between August 2019 and March 2020 by the first author. For the chair yoga participants, the questionnaire assessments were administered 1–2 weeks before, and again 1–2 weeks after, the chair yoga intervention. For control participants, the questionnaires were administered twice, once before and once after an 8-week period. Demographic information was collected, and cognitive capacity was assessed at the first data collection time point. Questionnaires were administered in the order PANAS, HADS, MFES, EQ-5L-3D. On occasion, data collection spanned more than one session if participants were tired. Assessments were conducted individually with participants in their own room. A relative or care home staff member was present for some assessments and assisted with answers to the demographic questions where necessary but did not assist with the answers to the questionnaires.

The chair yoga sessions took place at a convenient regular time of day, in a room that could seat at least 10 participants. Care home staff assisted participants moving to and from the room and were present during the sessions. A minimum attendance at 9 of the 16 sessions (55%) for a participant’s data to be included in the analysis was chosen to allow for medical appointments and other events likely to occur in this frail population that might disrupt regular attendance. Focus groups with participants and interviews with care home staff were held after the sessions to gather views on the feasibility of and engagement with the chair yoga, the results of which are reported elsewhere (Frampton et al., in preparation).

### Data Analysis

Differences between the groups and interactions across the data collection time points were analyzed using two-way analyses of variance (ANOVA) with the factors Group (chair yoga, control) and Time (pre-intervention, post-intervention). Where significant interactions were found, simple main effects analyses were used to compare groups at each time point, and each group across time. The significance level was set at *p* ≤ .05. Analyses were performed using the IBM SPSS statistics software, Version 29.

## Results

From the 28 participants in the chair yoga care homes who originally agreed to take part in the study, 2 withdrew before the chair yoga sessions began. In the control care homes, 20 residents originally agreed to take part and 4 withdrew before data collection began. No participants dropped out between collection of the baseline data and data collection after 8 weeks in the control group. Eight of the 26 participants who attended some chair yoga sessions did not meet the requirement to attend 55% of the sessions over the 8 weeks. Reasons for non-attendance included conflicting medical appointments, illness, medical emergencies, avoidance, symptoms relating to severe dementia including lethargy, and no recollection of previously doing the chair yoga. One participant who had completed more than 55% of the chair yoga sessions refused to answer the questions for the assessments. Data from 17 participants in the chair yoga group and 16 in the control group was therefore used in the analyses. Average attendance at the chair yoga sessions for the 17 participants who attended at least 55% of the classes was 80.5% (range: 56.2%–100%). No adverse events were reported during the chair yoga sessions.

Demographic characteristics of the chair yoga and control participants are shown in Table 2. Two participants in the control group were unable to undergo the ACE III assessments because of speech and sight issues. The mean ages of the two groups did not differ significantly. However, the chair yoga group was more cognitively impaired than the control group on the ACE III measure of cognitive capacity (independent *t* test: *t* (29) = −2.644, *p* < .05) and included more participants with scores in the severe dementia range (So et al., 2018).

**Table 2.** Demographic Characteristics.

	Chair Yoga	Control
Gender		
Male	3 (17.6%)	5 (31.3%)
Female	14 (82.4%)	11 (68.8%)
Age (years)		
Mean (*SD*)	89.5 (6.19)	89.0 (5.99)
Range	81–101	81–100
80–89	10	8
90–99	5	7
100–100+	2	1
Yoga: Experience prior to the study		
*N* =	5 (29.4%)	7 (43.8%)
Exercise: Typical per week		
<30 minutes	9	7
1–2 hours	4	5
>2 hours	4	4
Dementia diagnosis	9 (53%)	11 (69%)
ACE III score		
Mean score (*SD*)	49.4 (20.0)*	65.6 (12.5)
>=88 (no impairment)	0	1
67–87 (cognitive impairment)	3	6
54–66 (mild dementia)	6	4
37–53 (moderate dementia)	4	3
0–36 (severe dementia)	4	0

*Note.* *p < .05, mean ACE III score for chair yoga group (*N* = 17) compared with control group (*N* = 14).

**Figure 2.**
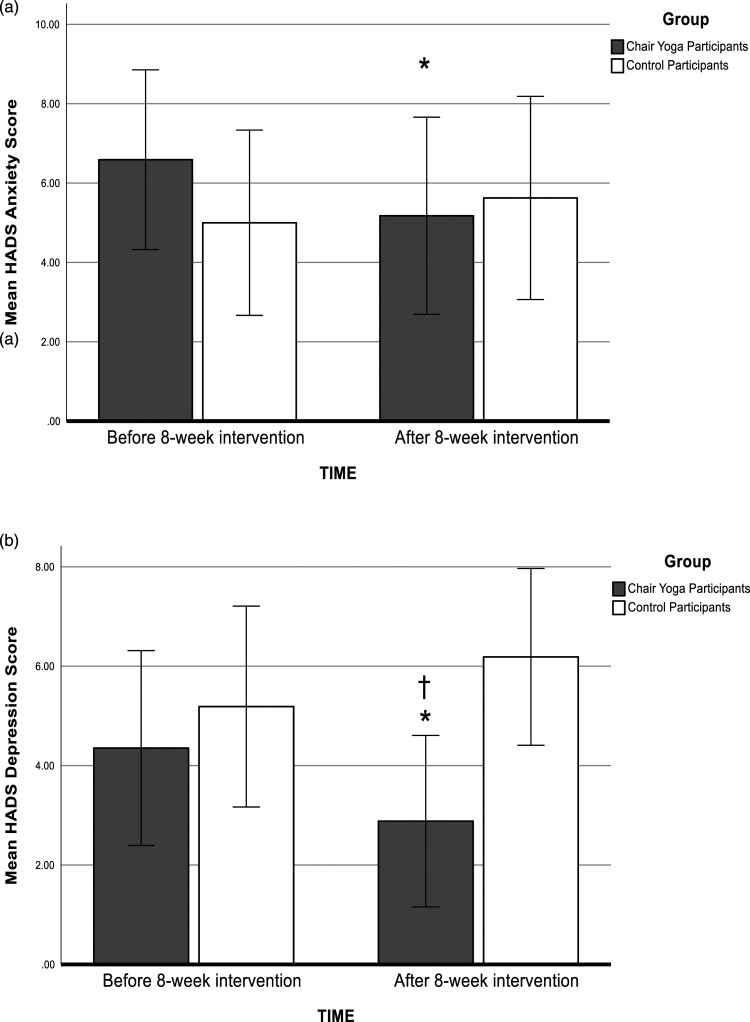
Anxiety and depression scores. Mean HADS (a) Anxiety and (b) Depression Scores as a function of intervention and time. *p < .05 after compared with before the intervention. †*p* < .05 chair yoga compared with control group. Error bars represent +/−2 standard error.

Mean HADS anxiety scores before and after the chair yoga intervention and at equivalent time points for the control participants are shown in Figure 2(a). A two-way ANOVA revealed a significant group (chair yoga/control) * time (pre/post) interaction (*F* (1,31) = 4.53*, p* = .04, partial ƞ^2^ = 0.128). Chair yoga participants were significantly less anxious post-intervention (*p* < .05), whereas anxiety levels were unchanged in the control participants. There were no significant main effects of group or time.

Figure 2(b) shows the mean HADS depression scores before and after the chair yoga intervention and at equivalent time points for the controls. A two-way ANOVA revealed a significant group (chair yoga/control) * time (pre/post) interaction (*F* (1,31) = 9.61, *p* = .004, partial ƞ^2^ = 0.237). Chair yoga participants were significantly less depressed post-compared with pre-intervention (*p* < .05), whereas depression levels were unchanged in the control participants measured at the equivalent time point. Chair yoga participants were also significant less depressed compared with the controls post-intervention (*p* < .05). There were no significant main effects of group or time.

**Figure 3.**
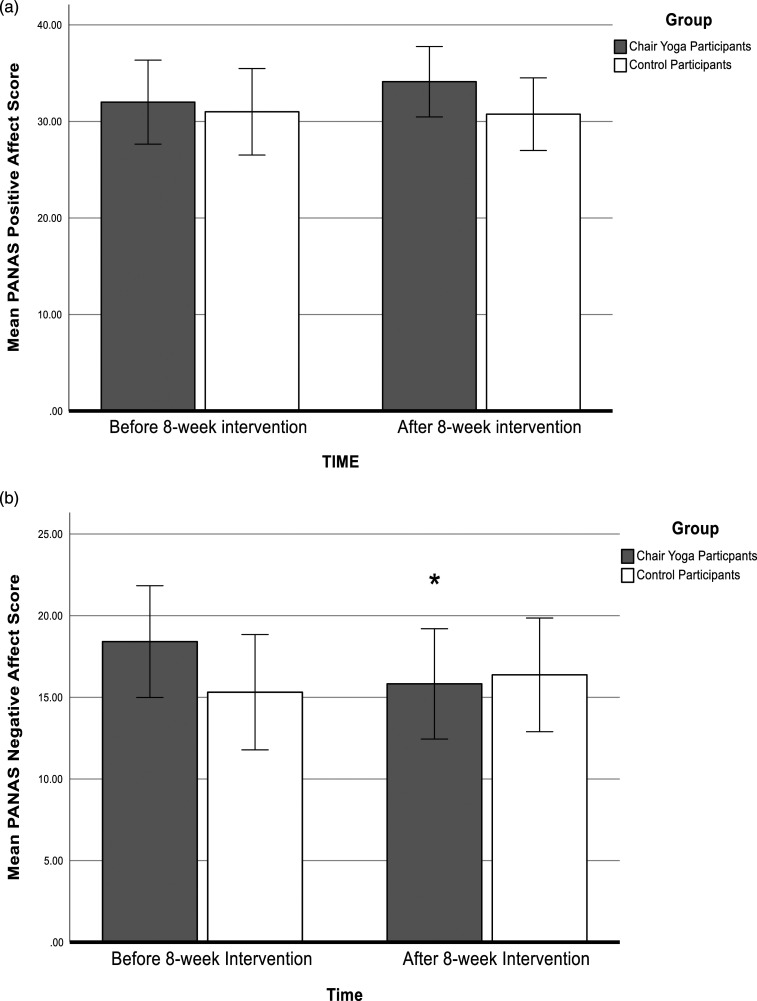
Positive and negative affect. (a) Mean PANAS Positive Affect Scores and (b) Mean PANAS Negative Affect Scores as a function of intervention and time. *p < .05 after compared with before the intervention. Error bars represent +/−2 standard error.

For positive affect on the PANAS, the group (chair yoga/control) * time (pre/post) interaction was not significant (*F* (1,31) = 1.73, *p* = .20), and there were no significant main effects of group or time (Figure 3(a)).

Negative affect mean scores on the PANAS decreased in chair yoga participants after compared with before the chair yoga sessions (Figure 3(b)). A two-way ANOVA revealed a significant group (chair yoga/control) * time (pre/post) interactions for negative affect (*F* (1,31) = 6.07, *p* = .02, partial ƞ^2^ = 0.164). Chair yoga participants were significantly less negative after compared with before the chair yoga intervention (*p* < .05), whereas negative affect scores of the control participants were unchanged 8 weeks after the baseline assessment. There were no significant main effects of group or time for negative affect. Changes in HADS and PANAS scores over the two time points did not depend on levels of cognitive impairment (supplemental material).

Mean scores on the Modified Falls Efficacy Scale for the chair yoga and control participants before and after the chair yoga sessions or at the equivalent time points are shown in Table 3. Data could not be collected for one chair yoga participant due to severe cognitive impairment and from one control participant at the second time point due to being bedbound. Baseline and post-intervention scores after 8 weeks did not differ for either group on this measure. A two-way ANOVA revealed no significant group * time (pre/post) interaction (*F* (1,29) = 2.81, *p* = .10), and no main effects of either group or time.

**Table 3.** Fear of Falling (MFES) and Health-Related Quality of Life (EQ-5D-3L).

	Chair Yoga		Controls	
	Pre	Post	Pre	Post
MFES				
mean (*SD*)	7.647 (2.276)	8.052 (2.300)	7.935 (2.779)	7.736 (2.859)
EQ-5D-3L				
Index mean (*SD*)	0.677 (0.230)	0.748 (0.141)	0.676 (0.352)	0.637 (0.385)
VAS mean (*SD*)	70.19 (20.75)	72.81 (23.59)	64.37 (29.68)	68.13 (21.69)
Better (%)		6 (37.5)		4 (25.0)
Same (%)		8 (43.8)		7 (43.8)
Worse (%)		2 (12.5)		5 (31.3)

For HRQoL, two-way ANOVAs revealed no significant group (chair yoga/control) * time (pre/post) interactions for the EQ-5D-3L index scores (*F* (1,30) = 1.85, *p* = .18) or the EQ-5D-3L visual analog scores (*F* (1,30) = 0.03, *p* = .87). There were no main effects of either group or time for EQ-5D-3 L index or EQ-5D-3L visual analog scores (Table 3).

## Discussion

The present study provides evidence that accessible chair yoga can be delivered in residential care homes and is associated with improved mental wellbeing of participants, including reduced anxiety and depression, and improved mood. HRQoL and fear of falling were unchanged following the chair yoga sessions.

Good attendance (80.5%) by the 17 participants who met the 55% attendance requirement for inclusion indicates delivering accessible chair yoga in residential care homes is feasible. However, about a third of those who took part in some sessions did not meet this attendance requirement. The most common reasons for non-attendance were medical, including illness and doctor’s appointments. Occasionally, participants refused to attend on the day or denied attending previously. Previous chair yoga studies with community-based older populations used a 75% attendance requirement (Furtado et al., 2016; Park et al., 2020). Achieving this level of attendance may not be feasible in care homes given the frailty of many residents. Saravanankumar et al. (2014) used 50% attendance as the feasibility criterion for a tai chi and yoga intervention for older participants living in residential care. It is encouraging that despite the attendance requirement of only 55% in the present study, significant improvements were observed on measures of mental wellbeing. This suggests care home residents may benefit from attending chair yoga sessions even if circumstances prevent them from attending every session offered. Further aspects of feasibility will be explored in the qualitative analysis of the focus groups and interviews to be reported elsewhere (Frampton et al., in preparation).

The finding that depression reduced significantly in the chair yoga group is in accord with previous reports of yoga effects in older populations (Bonura & Tenenbaum, 2014; Krause-Sorio et al., 2022; Park et al., 2020). Systematic reviews have also identified positive effects of yoga interventions on depression in healthy older people (Chobe et al., 2020; Sivaramakrishna et al., 2019). The novel finding in the present study is that accessible chair yoga delivered in care homes can also positively impact depression.

Reduced anxiety following yoga has been reported in healthy older adults (Bonura & Tenenbaum, 2014) and adults with subjective cognitive decline (Krause-Sorio et al., 2022), but two studies for participants with dementia reported no change in anxiety (Litchke et al., 2012; Park et al., 2020). A meta-analysis considering results of four yoga studies with older adults found a non-significant trend for reduced anxiety (Sivaramakrishnan et al., 2019), as did a chair yoga study (Galantino et al., 2012). Therefore, the evidence to date on yoga and anxiety is inconclusive. The finding here that anxiety reduced following the chair yoga intervention adds to the evidence that yoga may be a useful activity to help care home residents deal with anxiety.

Few yoga intervention studies for older adults have measured mood separately from depression. Oken et al. (2006) reported no change in mood measured by the Profile of Mood States following a 6-month yoga intervention for healthy older adults. The present study found significantly reduced negative affect scores on the PANAS in the chair yoga group, whereas affect scores were unchanged in the control group. This result is encouraging that chair yoga can improve mood in care home residents, although further studies that specifically assess mood independently from depression would be useful.

Fear of falling was unchanged after compared with before the chair yoga and did not differ significantly from the control group. This result contrasts with decreased fear of falling reported by Furtado et al. (2016) and Galantino et al. (2012) in older adults after chair yoga. However, it is not clear whether these decreases were specific to the chair yoga as fear of falling also decreased in the control group in Furtado et al. (2016), and there was no control group in Galantino et al. (2012). A meta-analysis by Sivaramakrishnan et al. (2019) found no significant effect of yoga on the fear of falling in older adults. The intention in the present study had been to measure actual falls in the months before and after the chair yoga sessions using the falls data routinely recorded by care home staff. However, falls data was not available due to the pressures on care home staff during the COVID-19 pandemic that commenced in the UK shortly after the other data had been collected.

HRQoL scores post-intervention in the chair yoga group did not differ significantly from the control group. Park et al. (2020) reported improved HRQoL in adults with dementia who took part in chair yoga compared with a music intervention. A meta-analysis also found positive effects of chair yoga on HRQoL in older adults (Tulloch et al., 2018). It is not clear why the present study result differs from these previous reports, although it may be that the majority of participants in previous studies lived in the community rather than in residential care. For the care home residents in the present study, scores were similar to population norms (EuroQol, 2022). Life experiences may have seemed similar from day to day and therefore HRQoL questionnaire responses remained essentially unchanged.

The present study has both strengths and limitations. A strength is that the chair yoga was delivered, and data collected, in the care homes where the participants were resident. While many previous studies have investigated potential benefits of yoga for healthy older adults and those with dementia or MCI (Bhattacharyya et al., 2021; Brenes et al., 2019; Karamcoska et al., 2023; Wang et al., 2022), relatively few have undertaken studies with residents in care homes (Fan & Chen, 2011; Hariprasad et al., 2013; Saravanakumar et al., 2014). Evidence regarding potential benefits of chair yoga delivered in a care home environment provides useful information for managers deciding what activities to provide for residents. Importantly, the present study included participants with a range of cognitive capacities typical of care home populations, using the ACE-III to characterize cognitive ability rather than relying on a dementia diagnosis, as dementia may be undiagnosed in some care home residents (Aldridge et al., 2023). The present findings are encouraging that despite the moderate to severe dementia in several participants (Table 1), depression, anxiety, and mood improved in the chair yoga group overall, although the results should be interpreted with caution given the measures have not been validated for use with people living with dementia and may be affected by dementia severity (Stott et al., 2017). A limitation is the present study was not designed to compare effects across different levels of dementia. Rather, the purpose was to explore the feasibility and potential effectiveness of chair yoga in a typical care home population, including individuals at various stages of dementia.

Use of the EPYQ to characterize the chair yoga intervention is a strength of the present study. Standardizing descriptions of yoga interventions by using the EPYQ is recommended to facilitate comparisons between studies (Park et al., 2018). The inclusion of a control group is a further strength that not all previously published chair yoga studies have employed (Galantino et al., 2012; Litchke et al., 2012; McCaffrey et al., 2014). The usual care comparison in the present study controlled for any changes due to the passage of time or repeated testing, rather than the chair yoga intervention per se. A limitation of the present study is the absence of an active control group, for example, chair-based exercise or music that would be needed to determine which aspects of the chair yoga contributed to the observed changes. Future studies will ideally include both a passive (usual care) and active control group for comparison with the yoga intervention (Bouega, 2020).

Further limitations of the present study were that the allocation to groups was not randomized, and data collection was not blind with respect to groups. Blinding participants to an intervention such as chair yoga is difficult since inevitably participants are aware they have taken part, unlike a drug study that can disguise a placebo control. An equally plausible intervention would be needed as the active control. The approach taken in the present study was to use a cluster-design, as used in other yoga studies in care homes, in which participants were allocated to groups depending on the care home in which they lived (Fan & Chen, 2011; Hariprasad et al., 2013; Park et al., 2020). This design has the advantage of reducing the risk of cross contamination, for example, potential disappointment if participants realize they are not in the active arm of the study. Future studies should consider using cluster-designs in which care homes are allocated randomly to the different arms of the study, and data collection is undertaken by researchers unaware of the allocation and therefore blind to the intervention.

In common with most yoga intervention studies, measures in the present study were taken in the weeks immediately before and after the chair yoga intervention. Further studies are required to establish whether the observed changes would persist in the weeks and months after the chair yoga sessions, or whether continued participation was necessary for sustained improvements. Similarly, further studies are required to establish the minimum effective ‘dose’, for example, whether the twice weekly sessions used here were necessary or whether a single session each week would have been sufficient.

In conclusion, this study provides preliminary evidence that older adults, including those living with dementia, can benefit from chair-based yoga sessions delivered in the care home in which they are resident. The benefits were seen even though residents were not always able to attend all the twice weekly sessions, for example, because of medical appointments. The focus of the present study was on mental health, with depression, anxiety, and mood improving in the chair-yoga but not the control group. Future randomized controlled trials, including an active control group, are required to address further questions about the potential benefits of chair yoga for care home residents, including on falls and other physical measures, and whether continued participation is required to sustain benefits in the longer-term.

## Supplemental Material

Supplemental Material - Reduced Anxiety and Depression and Improved Mood in Older Adults Living in Care Homes After Participating in Chair Yoga Supplemental Material for Reduced Anxiety and Depression and Improved Mood in Older Adults Living in Care Homes After Participating in Chair Yoga by Keeley Frampton, Liz Oppedijk, Rebecca Hadley, and Lucy E. Annett in Journal of Applied Gerontology.
