# Supplementary material for: Reduced Anxiety and Depression and Improved Mood in Older Adults Living in Care Homes After Participating in Chair Yoga
Source: J Appl Gerontol. 2024 Mar 24;43(10):1408–18. doi: 10.1177/07334648241241298 (PMC11370153; doi:10.1177/07334648241241298)
Supplement: Supplemental Material - Reduced Anxiety and Depression and Improved Mood in Older Adults Living in Care Homes After Participating in Chair Yoga [file sj-pdf-1-jag-10.1177_07334648241241298.pdf]

Supplementary Figure.

A.

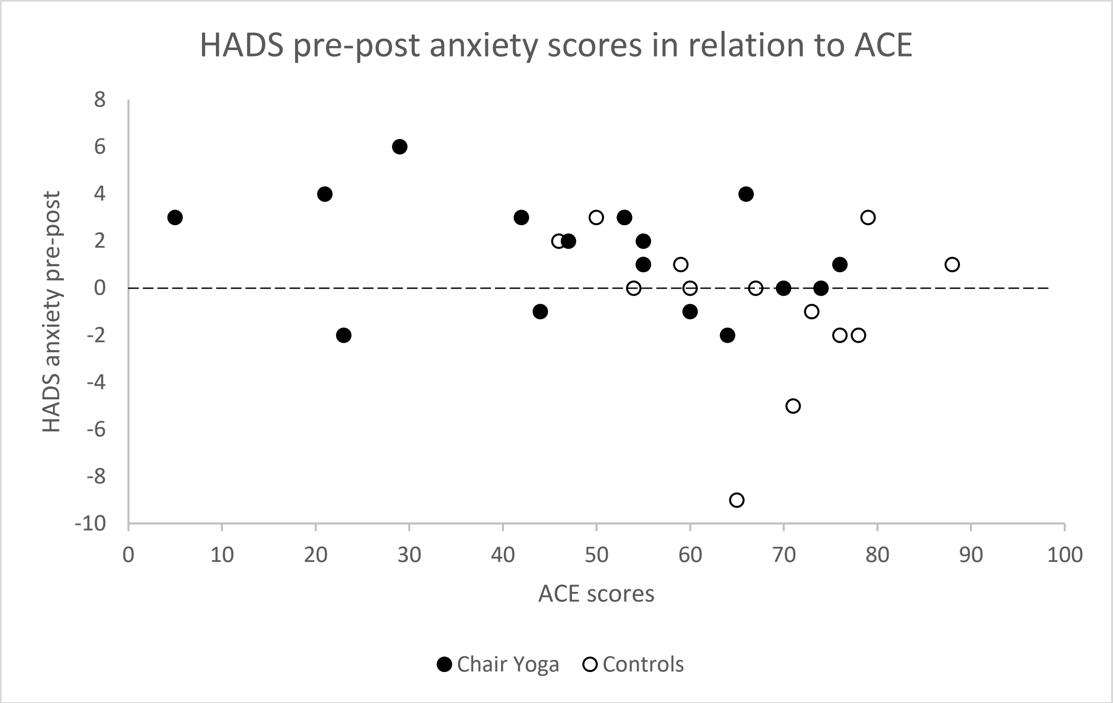

B.

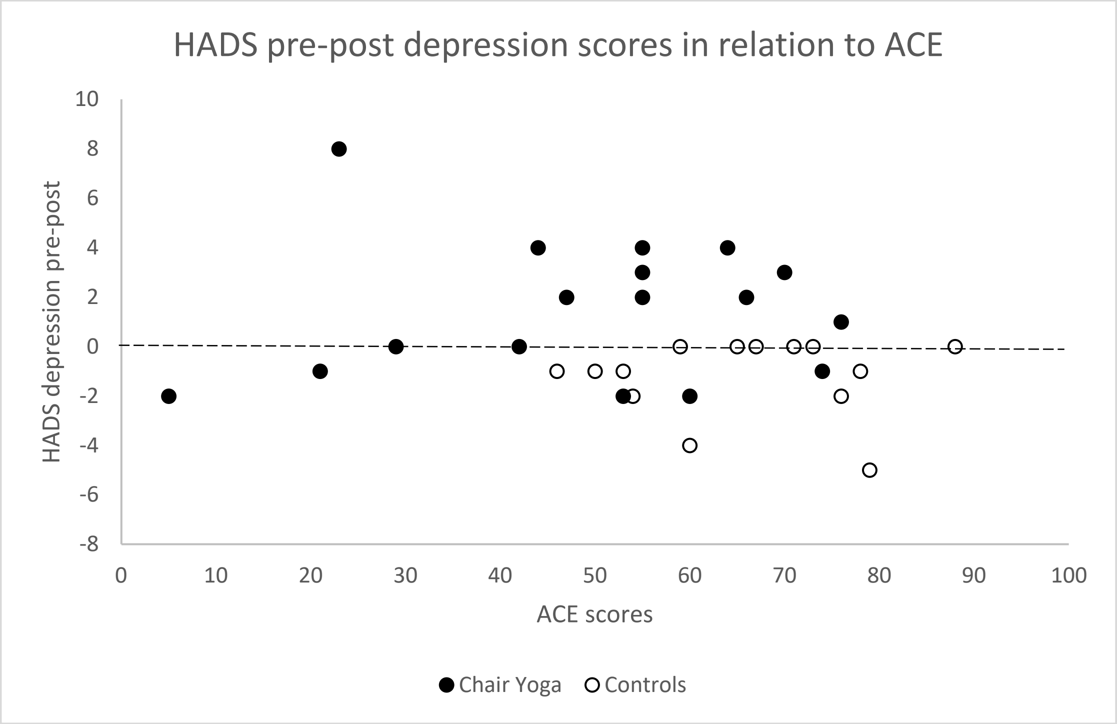

C.

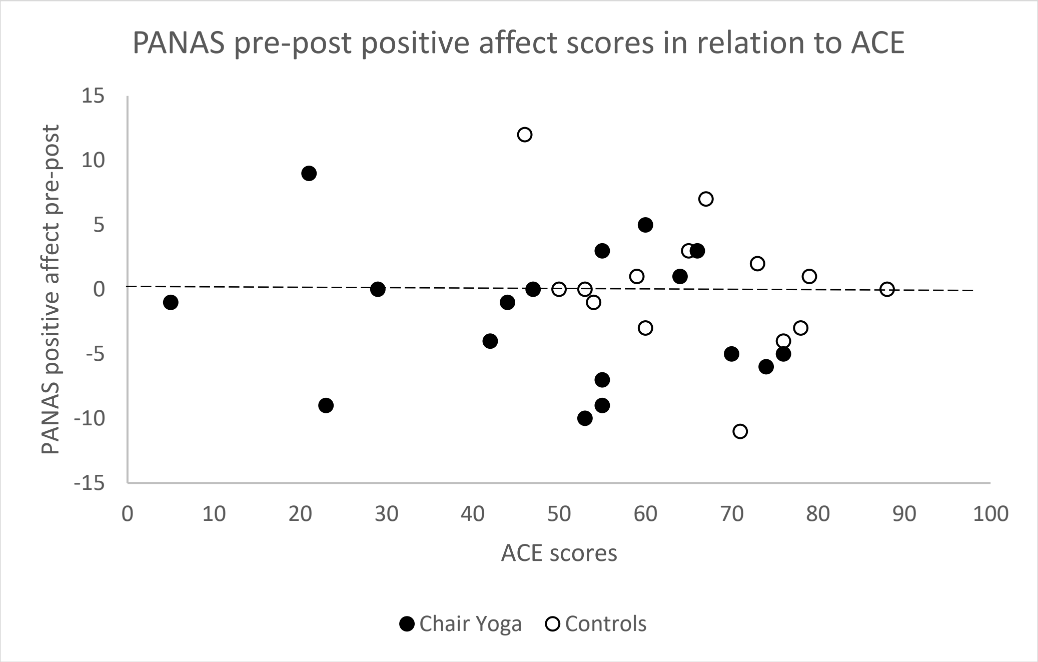

D.

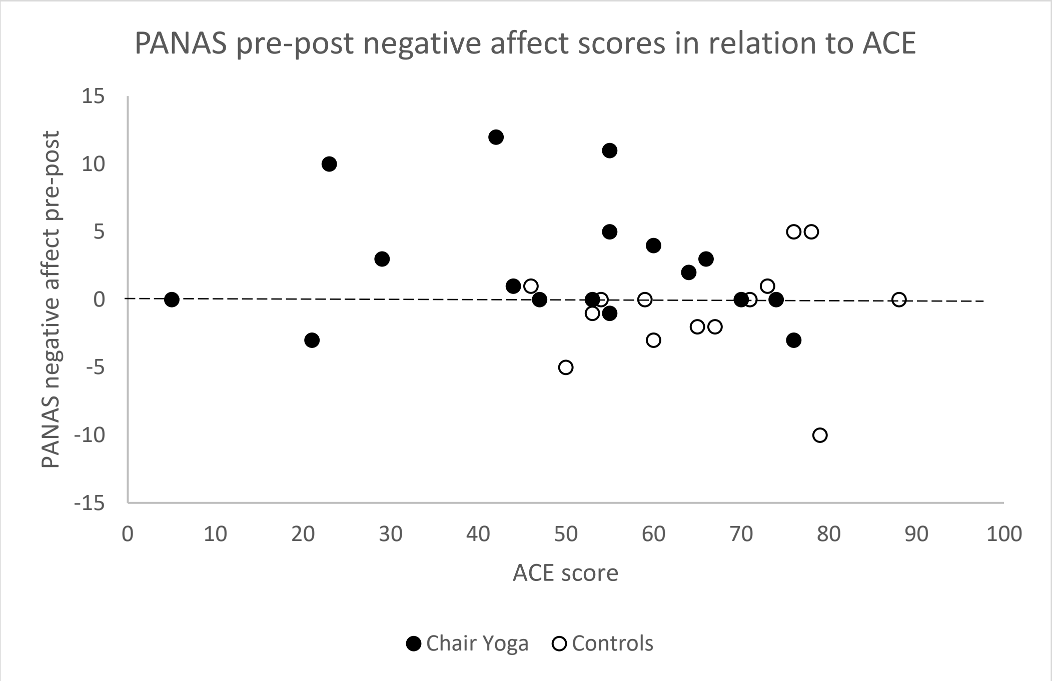

Fig. S1. The change in scores (pre-intervention minus post-intervention) for A) HADS anxiety, B) HADS depression, C) PANAS positive affect, D) PANAS negative affect, plotted against ACE III cognitive impairment scores for individual chair yoga (●) and control (○) participants. Note: low ACE scores indicate greater cognitive impairment. Scores above the dashed line in A indicate less anxiety, in B less depression, and in D less negative affect, whereas scores below the dashed line in C indicate more positive affect on the second (post-intervention) compared with the first (pre-intervention) test.
